# Highly acidic pH facilitates enamel protein self-assembly, apatite crystal growth and enamel protein interactions in the early enamel matrix

**DOI:** 10.3389/fphys.2022.1019364

**Published:** 2022-12-08

**Authors:** Youbin Zhang, Tianquan Jin, Weiying Zhu, Mirali Pandya, Gokul Gopinathan, Michael Allen, David Reed, Timothy Keiderling, Xiubei Liao, Thomas G. H. Diekwisch

**Affiliations:** ^1^ Department of Oral Biology, University of Illinois at Chicago, Dallas, Illinois, United States; ^2^ Department of Chemistry, University of Illinois at Chicago, Chicago, Illinois, United States; ^3^ Center for Craniofacial Research and Diagnosis, Texas A and M College of Dentistry, Dallas, Texas, United States; ^4^ Department of Medicine, University of Chicago, Chicago, Illinois, United States; ^5^ Department of Biochemistry, University of Illinois at Chicago, Chicago, Illinois, United States

**Keywords:** enamel, amelogenin, acidic, apatite crystals, electron microscopy

## Abstract

Tooth enamel develops within a pH sensitive amelogenin-rich protein matrix. The purpose of the present study is to shed light on the intimate relationship between enamel matrix pH, enamel protein self-assembly, and enamel crystal growth during early amelogenesis. Universal indicator dye staining revealed highly acidic pH values (pH 3–4) at the exocytosis site of secretory ameloblasts. When increasing the pH of an amelogenin solution from pH 5 to pH 7, there was a gradual increase in subunit compartment size from 2 nm diameter subunits at pH 5 to a stretched configuration at pH6 and to 20 nm subunits at pH 7. HSQC NMR spectra revealed that the formation of the insoluble amelogenin self-assembly structure at pH6 was critically mediated by at least seven of the 11 histidine residues of the amelogenin coil domain (AA 46–117). Comparing calcium crystal growth on polystyrene plates, crystal length was more than 20-fold elevated at pH 4 when compared to crystals grown at pH 6 or pH 7. To illustrate the effect of pH on enamel protein self-assembly at the site of initial enamel formation, molar teeth were immersed in phosphate buffer at pH4 and pH7, resulting in the formation of intricate berry tree-like assemblies surrounding initial enamel crystal assemblies at pH4 that were not evident at pH7 nor in citrate buffer. Amelogenin and ameloblastin enamel proteins interacted at the secretory ameloblast pole and in the initial enamel layer, and co-immunoprecipitation studies revealed that this amelogenin/ameloblastin interaction preferentially takes place at pH 4—pH 4.5. Together, these studies highlight the highly acidic pH of the very early enamel matrix as an essential contributing factor for enamel protein structure and self-assembly, apatite crystal growth, and enamel protein interactions.

## Introduction

Geological minerals require extreme temperatures, pressures, or pH for their formation, as well as long time periods for their transformation from individual ions to organized minerals (Veis and Dorvee 2013; Pandya and Diekwisch 2019). In contrast, biological minerals form at ambient temperatures and pressures, leaving control over crystal growth to conditions of the surrounding environment, including pH and the organic matrix (Veis and Dorvee 2013; Pandya and Diekwisch 2019). The organic protein matrix exerts far-reaching control over growth and habit of biological minerals such as tooth enamel (Lowenstam 1981; Diekwisch et al., 1993; Rao and Cölfen 2016). In addition, acidic pH increases hydroxyapatite formation and solubility and facilitates apatite synthesis *in vitro*, while pH elevation promotes apatite crystal growth and maturation (Larsen and Nyvad 1999; Chen et al., 2006). It has also been suggested that at acidic pH, specific protein binding sites may exist on crystal surfaces that may be released by protonation, which would lower cationic charge on both crystal surface and ionic charge on the protein (Robinson et al., 2005). Together, these studies indicate that both the organic matrix and the microenvironmental pH play a significant role in the nucleation and maturation of biological apatite crystals.

To protect ameloblast cellular compartments, individual ions and proteins are separated throughout their transport through the ameloblast layer, only to result in a dramatic convergence of minerals and proteins in the secretory enamel matrix immediately adjacent to the secretory ameloblast pole (Diekwisch et al., 1995; Pandya et al., 2017). At the secretory ameloblast pole, calcium ions, phosphate ions and enamel proteins converse and interact for the first time to form the initial enamel crystals (Pandya and Diekwisch 2021). Most notably, the concurring deposition of calcium and phosphate ions into the matrix leads to an almost instantaneous formation of apatite crystals (Jokisaari et al., 2019; Pandya and Diekwisch 2021) prompting excess proton production (Smith 1998; Smith et al., 2005; Lacruz et al., 2017). It has been speculated that acidic conditions resulting from the excess presence of free protons would then be buffered either through the presence of amelogenins or *via* bicarbonates and through other buffer systems in the extracellular matrix (Ryu et al., 1998; Smith 1998; Lacruz et al., 2010; Bori et al., 2016; Lacruz et al., 2017). In recent years, several groups have identified numerous ameloblast expressed transmembrane proteins involved in the production and transport of biocarbonates, including CFTR, AE2a,b, NBCEI, Na-hydrogen exchanger-1, carbonic anhydrase 2, as well as solute carrier family members Slc26a3, Slc26a4, and Slc26a6 (Bronckers et al., 2016). These findings underscore the high number of protons surrounding forming enamel apatite crystals and the sophistication of buffering mechanisms present in the developing enamel matrix.

While highly acidic pH has not been considered to occur during physiological amelogenesis, the first reported synthetic generation of apatite nanorods was based on an aqueous hydroxyapatite solution titrated to pH 2.8, resulting in the precipitation of 200–400 nm long apatite crystals (Chen et al., 2006). Low pH has also been popular for biochemical studies of enamel proteins since the earliest days of enamel research. Burgess and MacLaren. (1965) and Fincham. (1968) performed electrophoretic studies in starch-urea and polyacrylamide gels at extremely acidic pH values such as 3.7 and 3.0 respectively, lauding the high electrophoretic resolution attainable under these conditions. Amelogenin structure studies (pH 4) also have been conducted at acidic pH (Delak et al., 2009; Jin et al., 2009; Zhang et al., 2011; Shaw et al., 2020) to facilitate structure determination under high solubility conditions, avoiding the aggregate effects that occur near physiological pH. Several authors have reported monomeric, disordered, or small-sized (3–7 nm diameter) amelogenin assemblies at pH values between 3.0 and 5.8, compared to the 15–20 nm supramolecular assemblies detected at physiological pH (Moradian-Oldak et al., 1998; Bromley et al., 2011; Fang et al., 2011; Beniash et al., 2012). Supporting the concept of an acidic pH promoting apatite crystals growth, it has been reported that growth rates and numbers of apatite ribbons were significantly higher at pH4.5 when compared to pH7.0 (He et al., 2011). In contrast to the dearth of knowledge about the role of pH during early amelogenesis, pH changes in maturation stage enamel have been well established ever since the visually striking experiments by Takano et al. (1988), Sasaki et al. (1991). These studies have established a correlation between alternate acidic (pH 5.8–6.0) and neutral (7.0–7.2) ameloblasts at the zones of ruffle-ended and smooth-ended maturation stage ameloblasts of unerupted calf incisors (Sasaki et al., 1991). Several others have pointed to the importance of pH regulation and buffering for ameloblast differentiation and amelogenesis during the maturation stage (Wiedemann-Bidlack et al., 2007; Tye et al., 2010; Wang et al., 2010; Lacruz et al., 2012).

The present studies were prompted by the detection of an acidic pH at the exosytosis site of secretory ameloblasts. Based on this initial finding we conducted studies to uncover how such highly acidic pH might affect enamel protein assembly, crystal growth, and enamel protein interactions. To verify our hypothesis, the pH of the early enamel matrix was tested by staining freshly prepared matrix. To determine the effect of pH on amelogenin self-assembly we have conducted a series of biochemical and physicochemical studies of individual components of the enamel protein matrix. These studies suggest that a highly acidic pH occurs at the exocytosis site of secretory ameloblasts and provide insights into the effects of pH on amelogenin self-assembly, calcium phosphate crystal growth on a nano- and macroscale, and amelogenin/ameloblastin interactions.

## Materials and methods

### pH detection on tissues using indicator solutions

Unerupted molar teeth from 4 months old cattle were chosen as a model to expose a fresh cut of sufficient size through the developing epithelial mesenchymal interface to perform macroscopic imaging of fresh tissue for pH analysis. For this study, jaws from 4 months old steers were obtained from Brown Packing, South Holland, IL, and impacted molar teeth were dissected using surgical tools. Molar teeth were then cut in half using a surgical blade and half molars immersed in Universal pH Indicator solution (Sigma, St. Louis, MO) for 10 min and gently rinsed with distilled water. Tissue surfaces were then covered with a coverslip, and the distribution of the pH indicator on the tissue section was imaged using light microscopy.

### Circular dichroism measurements

The recombinant mouse amelogenin protein solutions were prepared at a 0.1 mg/ml concentration in PBS, and a HCl solution was used to adjust the pH for each sample solution. Each sample solution was transferred into a 1 mm pathlength quartz cuvette (Starna, Inc.), and CD spectra were measured from 180 nm to 250 nm with a 50 nm/min scanning rate, 2 s response time, 1 nm bandwidth as the average of eight scans on a JASCO 810 spectrometer (Jasco, Inc.) at room temperature. All sample spectra were corrected by subtraction of the corresponding buffer spectrum.

### Attenuated total reflectance fourier transform infrared (ATR-FTIR) spectroscopy

This study used the same amelogenin protein solutions as employed for the CD studies. For measurements, the FTIR spectrometer (Bruker Vertex 80) was equipped with an ATR accessory fitted with a diamond crystal (PIKE MIRacle single reflection ATR). A volume of 40 μl of sample solution was loaded onto the crystal and dried with a mild flow of nitrogen gas to form a uniform thin film on the crystal surface. Sample absorbance spectra over the range 4500 cm^-1^ to 600 cm^-1^ were collected as an average of 1024 scans (10 kHz scan speed with a DTGS detector) and processed with 3-term Blackman-Harris apodization and zero filling of 2. Experiments were repeated 3 times. Background transmission spectra were collected on the empty ATR crystal surface under the same conditions and were used to compute the absorbance spectra.

### Atomic force microscopy

Atomic force microscope (AFM) studies were carried out using an extended MultiMode AFM (MMAFM) integrated with a NanoScope IIIa controller (Veeco Instruments, Santa Barbara, CA) and a Q-Control Module (nanoAnalytics, Muenster, Germany) as previously described (Jin et al., 2009). The MMAFM was equipped with a calibrated E-type piezoelectric scanner and a glass cell for fluid TappingMode AFM (both from Veeco). The silicon AFM cantilever/probe used in this study was rectangular in shape, 130 µm in length and 35 µm in width (NSC36, MikroMasch). The advertised typical force constant and resonant frequency of this cantilever/probe is 0.6 N/m and 75 kHz respectively. Nominal sharpness of the probe-tip end radius is ≤ 10 nm. The cantilever/probes were oscillated near 30 kHz at low amplitude for fluid tapping mode AFM. Fluid damping reduces the resonant frequency of rectangular AFM cantilevers in air by approximately 50%. The AFM substrate used for protein adsorption was Grade V5, Pelco mica (10 × 40 mm) purchased from Ted Pella (Redding, CA). The mica was freshly cleaved using adhesive tape prior to use. Stock solutions of 10–20 mg/ml amelogenin M179 in 40 mM Tris were mixed and stored at 4°C and analyzed by AFM. Stock solutions were diluted typically at 1:100 into the blank AFM imaging buffer (40 mM Tris) during scanning and adsorption to mica was monitored. The pH of the imaging solution was adjusted to pH 5.0, 6.0, or 7.0 using concentrated HCl and verified using a pH electrode. Typical AFM scan rates were 1.0–1.25 Hz for 512 data points x 256 lines. The AFM images were plane-fit to correct for background sloping errors.

### Transmission electron microscopy of amelogenin assembly at various pH values

To assess the dimensions and patterns of self-assembled amelogenins under changing pH, solutions of 10–20 mg/ml amelogenin M179 in DDW were adjusted to pH 5.0, pH 6.0 and pH 7.0, and allowed to air dry on 200 mesh carbon coated electron microcopy grids (Ted Pella, Redding, CA). TEM grids obtained from each individual group were quickly rinsed with DDW, blotted against filter paper, and air dried. Transmission electron microscopy was performed using a JEOL 1220 TEM as previously described (Pandya and Diekwisch, 2019).

### *In vitro* crystal growth studies on polystyrene plates

To mimic the dehydrating conditions in the developing enamel matrix in an *in vitro* model, approximately 2 ml of 2.5 mM CaHPO_4_ at varying pH (pH 3.0 to pH 7.0) in a 35 mm non-coated polystyrene plate was placed in a 37°C incubator and allowed to evaporate to enable crystal formation.

### Crystal growth studies on TEM grids

Hydroxyapatite crystal growth studies were performed as previously described (Jin et al., 2009). Briefly, carbon-coated gold TEM grids were immersed into either a pH 4 or pH 7 crystal growth solution. The pH of the crystal growth solution was adjusted by adding either 20 mM NH_4_OH or 2.5 mM CaHPO_4_ buffer to DDW. For crystal growth studies, 2.5 mM CaCl_2_ and 1.5 mM (NH_4_)_2_HPO_4_ were added to the solution (Jin et al., 2009), which was then incubated in a moisturized container at 37°C for 2.5 h. Subsequently, TEM grids obtained from each individual reaction step were quickly rinsed with DDW, blotted against filter paper, and air dried. Transmission electron microscopy was performed using a JEOL 1220 TEM as previously described (Pandya and Diekwisch, 2019).

### Nano-hydroxyapatite (HA) binding to N92 amelogenin protein

10 µg of N92 amelogenin protein were incubated in 200 µl of nano-hydroxyapatite solution (HA) (0.1 μg/μl) of varying pH (pH 3.0 to pH 8.0) at 37°C for 1 h. The pH of the HA solution was adjusted over the range of pH 3.0 to pH 8.0 using glacial acetic acid or ammonium hydroxide. After 1 h incubation on a shaker, protein-HA complexes were washed with excessive amounts of 1xPBS, three x times at 10.000 x g to remove non-specific binding. Adsorbed protein from HA was released using 100 µL of RIPA lysis buffer. 20 µL of each sample protein over the range of pH 3.0 to 8.0 were run on a 10% SDS-PAGE gel, transferred onto a PVDF membrane in a semi-dry blotting apparatus containing transfer buffer (25 mM tris-HCl, 40 mM glycine, 10% methanol) for 45 min at 75 mA. The PVDF membrane was blocked with 5% bovine serum albumin for 1 h at room temperature, incubated with a chicken amelogenin antibody at a concentration of 1:5,000 for 1 h. Thereafter, the membrane was washed with TBST three times for 10 min each and probed with a HRP-conjugated anti-chicken secondary antibody at a concentration of 1:2,000. HRP was detected using chemiluminescent substrate (Supersignal West Pico Chemiluminescent Substrate, Pierce). For densitometric analysis, band intensities of three replicate films were determined using Adobe Photoshop. Protein expression was quantified as band intensity corrected for protein loading based on pH 7.0 intensity for each corresponding band.

### NMR studies

Expression vectors pASK-43 (+) were purchased from The TAG company (Göttingen, Germany). The ^15^N labeled amide chloride and D_2_O (99.5%) was purchased from Cambridge Isotope Laboratories (Andover, MA). 400 MHz PCR tubes were obtained from Kontes (Vineland, NJ). The following peptides used in this study were synthesized at the UIC core facility: TRAP (mouse amelogenin amino acids 1–45) and NLRAP (the N-terminal portion of the mouse amelogenin amino acids 1–33). Other common regents were from Sigma Aldrich (St Louis, MO). The expression of and purification of the amelogenin N-terminus (N33) were described previously (Zhang et al., 2011). The final concentration of ^15^N enriched amelogenin for our NMR study was 1 mM. The peptides were dissolved into phosphate buffer (5 mM phosphate, 50 mM NaCl) at either pH 4.5 or pH 6.0. The beginning concentration for HSQC data acquisition using the labeled peptide (TRAP) was 0.1 mM and with the unlabeled peptide (N33) was 0.5 mM. 10% D_2_O was added to all the samples for the NMR study. NMR data collection was performed on a 900 MHz Bruker NMR at the UIC Core Facility at room temperature and at either pH4.5 or pH6.0. Standard 2D HSQC spectra were acquired and processed using NMRPipe (Delaglio et al., 1995).

### Transmission electron microscopy of developing mouse molar tooth organs subjected to various buffers and pH conditions

Three-day postnatal mouse molar tooth organs were prepared from postnatal mice after euthanization by CO_2_ inhalation according to the guidelines of the University of Illinois, Chicago Animal Care Committee. Following sacrifice, the mouse molars were immediately dissected and either immersed in phosphate buffer at pH 7 or in phosphate buffer at pH 4 or in citrate butter at pH 4 for 2 h. Following immersion in each buffer system for 2 h, tooth organs were fixed according to Karnovsky’s protocol as previously described (Diekwisch, 1998).

Citrate buffer was generated by mixing 0.5 g of citric acid monohydrate and 0.4 g of dibasic sodium phosphate in 1 L H_2_O, and the pH was adjusted to either 4.0 or 7.0 by varying the amount of citric acid. Phosphate buffer was generated by mixing 5.04 g (pH 4) or 0.5 g (pH 7) of anhydrous disodium hydrogen phosphate and 0.301 g of potassium dihydrogen phosphate in 1 L H_2_O. Freshly dissected 3 days postnatal mouse molar samples were then directly immersed into the buffer for 2 h and fixed thereafter in Karnovsky’s.

### Co-immunolocalization studies for amelogenin and ameloblastin in developing mouse molars

Molar teeth harvested from 3-day postnatal wild-type and amelogenin null mice were sacrificed according to the guidelines of the University of Illinois, Chicago Animal Care Committee, dissected, fixed in 10% buffered formalin, and prepared for paraffin histology. Paraffin sections were then subjected using immunohistochemistry as previously described (Luan et al. 2007; Walker et al. 2008). Following pre-treatment, sections were then incubated with affinity-purified polyclonal anti-amelogenin (Satchell et al., 2002) or anti-AMBN (Lu et al., 2013) antibodies at a dilution of 1:100 at 4°C overnight. Dual labeling of protein expression was performed with a Double Staining AEC/DAB kit (Abcam, Waltham, MA).

### Validation of amelogenin and ameloblastin interaction *via* co-immunoprecipitation

To probe whether amelogenin and ameloblastin interact on a protein level, amelogenin (N-terminal 92 amino acids, N92) and ameloblastin (full-length) constructs were generated. Briefly, amelogenin N92 cDNA was inserted into the pNTAP vector to generate the CBP-SBP-amelogenin fusion protein, and full length ameloblastin cDNA with a c-terminal FLAG tag was inserted in the pCDNA3 vector to generate the ameloblastin-FLAG fusion protein. *In vitro* translation fusion proteins were generated from each vector (N92 amelogenin and ameloblastin-FLAG) using the TNT^®^ Coupled Reticulocyte Lysate Systems (Promega, Madison, WI). Following *in vitro* translation, co-immunoprecipitation assays were performed to analyze the interaction between ameloblastin and amelogenin.

To pull down co-precipitated amelogenin, anti-FLAG agarose (Sigma, St. Louis, MO) beads were incubated overnight with both proteins or amelogenin only (as non-specific binding control) in modified PBS buffer (with 2 mM CaCl2, 1 mM MgCl2 at either pH4.0, pH4.5, or 5.0). After overnight incubation, beads were washed with PBS buffer 3 times, mixed with loading buffer to boil, and subjected to SDS-PAGE gel electrophoresis for Western blot using anti-CBP antibody (Abcam, Cambridge, MA) to detect amelogenin co-precipitation.

To pull down co-precipitated ameloblastin, streptavidin sepharose (GE Healthcare, Pittsburgh, PA) beads were incubated overnight with both proteins or ameloblastin only (as non-specific binding control) in modified PBS buffer (with 2 mM CaCl2, 1 mM MgCl2 at either pH4.0, pH4.5, or 5.0). After overnight incubation, beads were washed with PBS buffer 3 times, mixed with loading buffer to boil, and subjected to SDS-PAGE gel electrophoresis for Western blot using anti-FLAG antibody (Sigma, St. Louis, MO) to detect ameloblastin co-precipitation.

## Results

### pH indicator dyes revealed pH values between pH 3 to 4 at the exocytosis site of secretory ameloblasts

**FIGURE 1:**
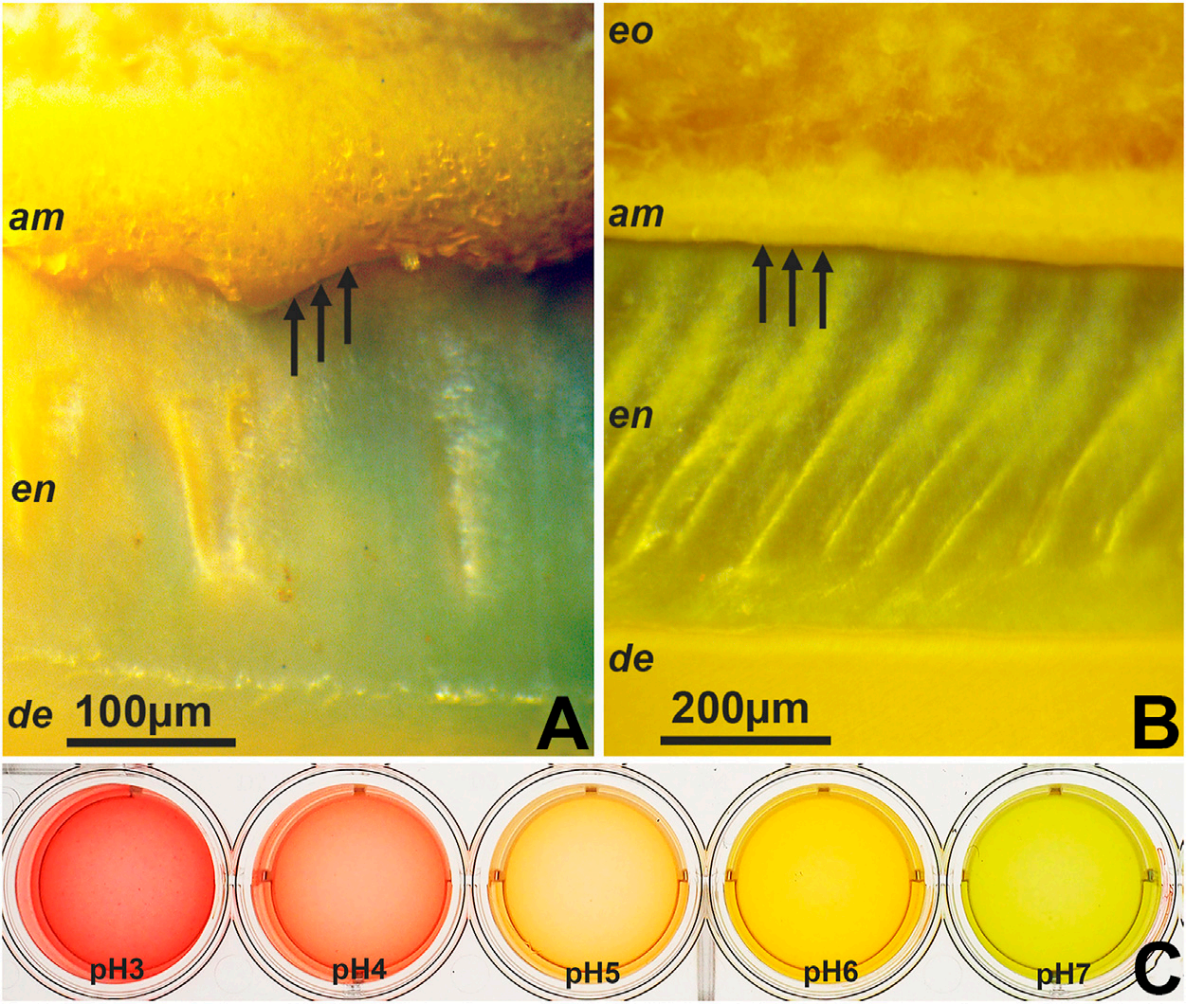
Areas of intense pH indicator staining in developing tooth enamel as revealed by pH indicator dyes. **(A,B)** pH distribution at the exostosis site of secretory ameloblasts and at the ameloblast/enamel interface during secretory stage enamel formation of freshly cut bovine molars. **(C)** Reference solutions prepared from pH3 to pH7 and stained with indicator dye. CL = cervical loop, am = ameloblasts, en = enamel, de = dentin, od = odontoblasts, eo = enamel organ.

Universal pH indicator dye (Sigma, St. Louis, MO) was used to determine the pH at the ameloblast/early matrix interface freshly cut bovine developing molar slices. Universal indicator staining of freshly cut bovine molar slices revealed a dark red stained band indicative of pH 3 right between the ameloblast layer and the enamel layer at the earliest secretory stage and during the maturation stage (Figure 1). These data suggest the presence of a highly acidic localized pH in the range between pH 3 and pH 4 at the exocytosis site of secretory ameloblasts (Figure 1).

### Increased amelogenin self-assembly and subunit diameter, as well as structural transformation between pH 5 to pH 8

**FIGURE 2:**
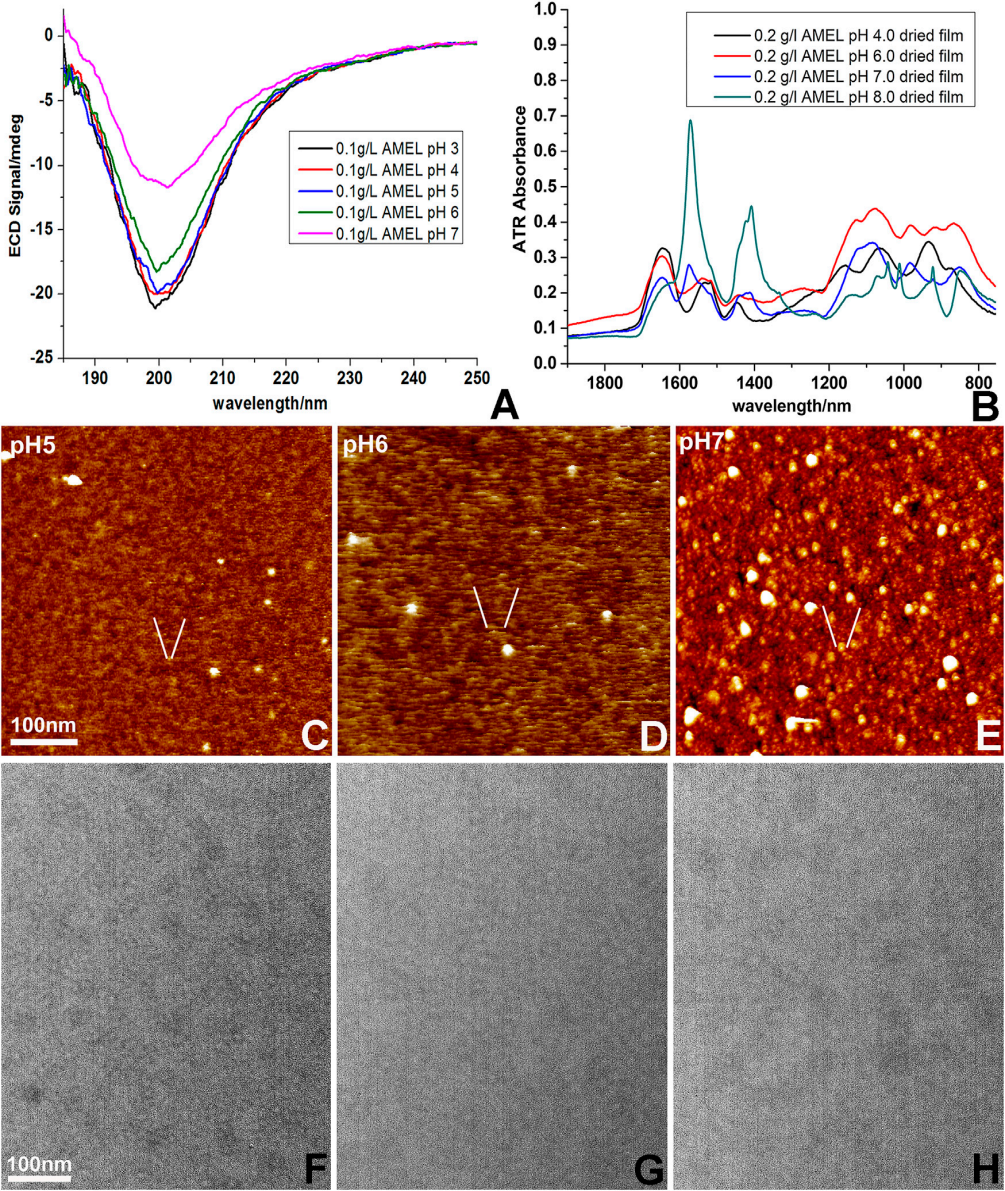
Changes in amelogenin self-assembly as a result of increasing pH. **(A)** Differences in electronic circular dichroism (ECD) spectra of amelogenin solutions as a result of increasing pH values from pH 3 to pH 7. **(B)** Dramatic changes in attenuated total reflection microscopy (ATR) spectra in amelogenin solutions ranging from pH 3 to pH 8. **(C–E)** Changes in amelogenin assemblies on freshly cut mica at pH 5, pH 6, and pH 7. **(F–H)** Increased diameter of amelogenin assemblies on carbon coated TEM grids prepared at pH 5, pH 6 **(E)**, and pH 7. A scale bar is provided for size comparisons.

CD spectra of amelogenins with increasing pH revealed a gradual loss of intensity for the 200 nm negative peak, suggestive of an increase in aggregation (Figure 2A). ATR spectra were typical of a disordered protein and displayed multiple changes with increasing pH, including a substantially elevated peak in the 1570 cm^−1^ region at pH 7.0 and pH 8.0, overlaying the amide II band and indicative of growth of a carboxyl bond, which may indicate an increasingly self-assembled and aggregated protein structure (Figure 2B).

Full length amelogenins were imaged between pH 5.0 and 7.0 on freshly cleaved mica and on coated electron microcopy grids using atomic force microscopy and transmission electron microscopy. At pH 5.0, atomic force microscopy revealed 2–3 nm subunits dispersed throughout the amelogenin solution while at pH 7.0, the mica surface was covered with typical 20 nm diameter spherical subunits. Remarkably, at pH 6.0 subunits consisted of 3 nm × 10 nm elongated stretches (Figures 2C–E). In contrast, subunit dimensions of amelogenin assemblies on carbon coated electron microscopy grids simply increased from 10 nm subunit sizes at pH 5.0–20 nm subunit sizes at pH 7.0 (Figures 2F–H). Together, these data suggest that during aggregation and self-assembly with increasing pH, amelogenin subunit dimensions increase in size, feature increased chemical bonds, and undergo unique intermediary configurations that may aid crystal growth.

### Changes in pH greatly affected the histidine rich region of the amelogenin coil domain (H47-H69) while the non-LRAP portion of the TRAP motif (AA34-45) was the key sequence motif for amelogenin self-assembly at pH 6

**FIGURE 3:**
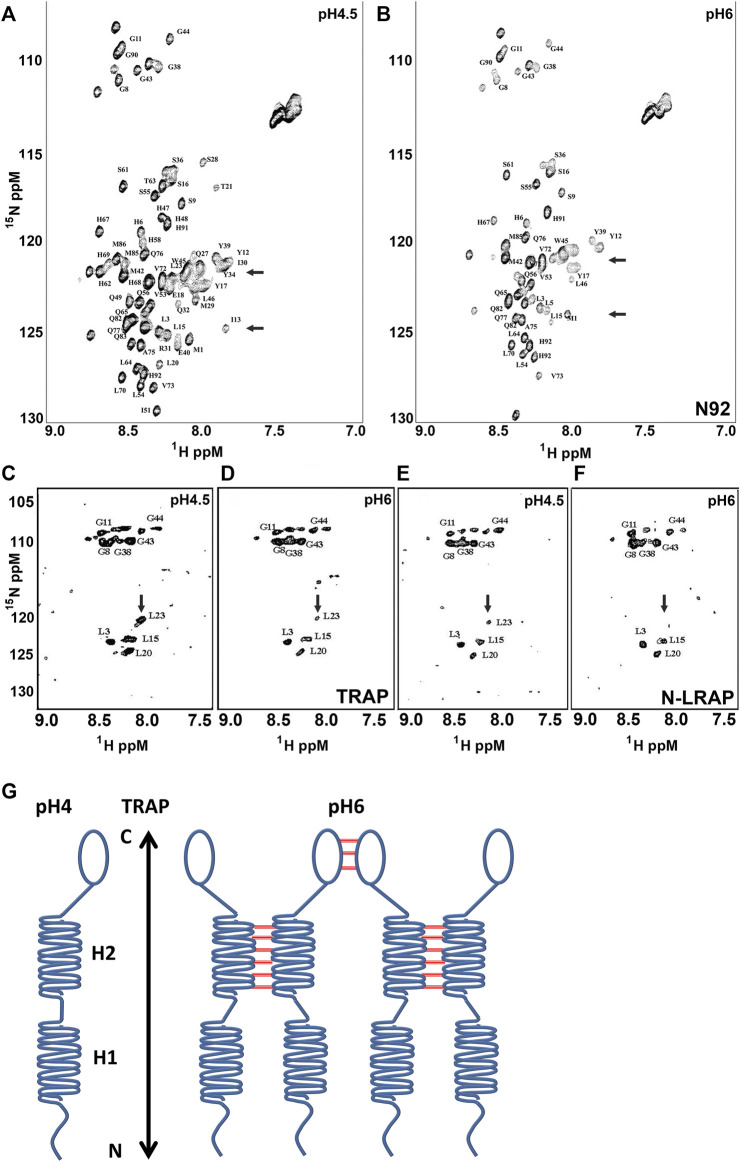
N-terminal amelogenin interactions at pH 4.5 and pH6. Figures A and B are HSQC spectra of N15 enriched Amelogenin (N92) at pH 4.5 **(A)** and pH6.0 **(B)** respectively. **(C,D)** are HSQC spectra of chemically synthesized amelogenin TRAP peptide (N1-45) with ^15^N-enriched Gly (G8, G11, G38, G43 and G44) and Leu (L3, L15, L20 and L23) at pH 4.5 **(C)** and pH 6.0 **(D)** respectively. **(E,F)** are HSQC spectra of the selected labeled TRAP peptide in the presence of a short peptide containing the first terminal 33 amino acid residues (N33, the N-terminal portion of LRAP) at pH 4.5 **(E)** and pH6.0 **(F)**. **(G)** is a model representing proposed intermolecular interactions between amelogenin molecules, with tight intermolecular bonds between the H2 helices in the non-LRAP TRAP region (AA34-45) and weak interactions at the amelogenin C-terminus. The H1 helices in the LRAP region did not feature any notable interactions.

Previous studies from our laboratory identified the amelogenin N-terminus as the major site for amelogenin self-assembly interactions (Zhang et al., 2011). To further query amelogenin NMR spectra for the effect of pH changes on N-terminal amelogenin interactions at low and high pH we acquired HSQCs of Amel-N (AA1 to 92) at pH 4.5 and pH 6 (Figure 3A). Prepared in solution, Amel-N was a monomer at pH 4.5 while there was a visible precipitate at pH 6.0, which did not interfere with the acquisition of HSQC spectra. When compared to the spectrum obtained at pH 4.5, a number of resonances mainly between E18 to H69 were either weakened or no longer detectable at pH 6.0, including E18, T21, L20, L23, Q27, M29, I30, Y34, H47, H48, H58, H62, H67, H68, and H69. This list includes seven of the nine histidines in the amelogenin N-terminal N92 fragment (H47, H48, H58, H62, H67, H68, H69), which comprises approximately half of all amelogenin histidines. The histidines in position 91 and 92 were little affected as residues at the amelogenin N92 N-and C-termini were not perturbed by changes in pH. Amelogenin contains four more histidines that were outside of the N92 fragment studied here. In summary, these HSQC spectra identified two possible interaction domains, 1) the histidine-rich region between amino acids 47—69 and 2) portions of the TRAP domain.

To further narrow down possible interaction sites within the TRAP domain, two peptides were synthesized, a TRAP peptide which contained the N-terminal 45 amelogenin residues and an Amel-N33 peptide containing the N-terminal 33 amelogenin residues. While the Leucine and Glycine of the TRAP peptide residues were ^15^N enriched, the N33 peptide (N-terminus of LRAP) did not contain isotopically enriched residues. Unlabeled TRAP peptide was retained for interaction studies with N-LRAP (Figures 3E,F). HSQC spectra for interactions between TRAP peptides (Figures 3C,D) and the unlabeled N33 with the labeled TRAP peptides (Fig. E, F) were acquired at pH 4.5 (Figures 3C,E) and pH 6 (Figures 3D,F), respectively. Both peptides were entirely soluble at pH 4.5. At pH 6 the TRAP peptide formed self-assembly precipitates similar to the ones observed with the N92 peptide, while the LRAP peptide did not. The soluble fraction of the TRAP peptide demonstrated a similar but slightly weakened resonance spectrum at pH 6 compared to the spectrum at pH 4.5 (Figure 3D vs.Figure 3C). In the N-LRAP peptide/TRAP interaction experiment, HSQC spectra of the selectively labeled TRAP peptide were further reduced, especially at L23 (Figure 3F vs. Figure 3E). At pH 6.0, the two leucine amino acids in positions L20 and L23 were no longer detected in the N92 spectrum while they were recognized in the TRAP spectrum (Figure 3B vs. Figure 3D), suggesting that interactions only occured in the full-length amelogenin and not in between individual amelogenin peptides.

1D homonuclear experiments were conducted to verify the effect of intermolecular interaction between N33 (N-terminus of LRAP) peptides. At pH 6.0 the 1D spectrum displayed a substantially increased line width when compared to pH4.5, typical for an aggregated peptide (data not shown). Together, these data indicate that the first 33 amino acid residues at the amelogenin N-terminus (N33) interacted with each other without forming large, precipitated nanostructures, while the C-terminus of the TRAP sequence (AA34-45) played a critical role in the formation of insoluble nanostructures at pH 6 (Figure 3G).

### Preferred nucleation and elongation of apatite crystals at acidic pH and preferred apatite binding of the N-terminal amelogenin between pH 3 and 6 vs. pH 7 and pH 8

**FIGURE 4:**
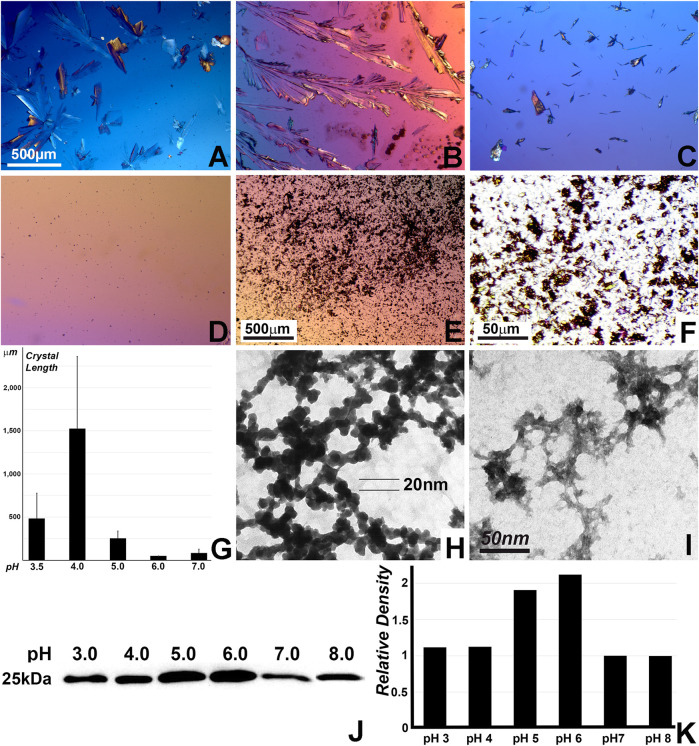
Effect of pH on calcium phosphate crystal growth and on the binding of the N-terminal amelogenin N92 to nano-hydroxyapatite. **(A–F)** Micrographs of calcium phosphate crystals grown on non-coated polystyrene plates at pH values from pH3 to pH7. **(F)** is a ten-fold enlarged view of **(E)** illustrating individual crystal shapes. **(G)** Average crystal length comparison based on the calcium phosphate growth study on polystyrene plates at pH values from pH3 to pH7 as performed in **(A–F)**. **(H,I)** Calcium phosphate crystal growth studies on carbon-coated TEM grids without the presence of protein. **(H)** calcium phosphate crystal growth at pH four and **(I)** calcium phosphate crystal growth at pH 7. The 50 nm scale bar in **(I)** is provided as a reference for both electron micrographs. **(J)** Nano-hydroxyapatite binding to N92 N-terminal amelogenin. For this study, N92 N-terminal amelogenin proteins were incubated at incremental pH values ranging from pH3 to pH8, adsorbed to hydroxyapatite, washed in lysis buffer, transferred on a PVDF membrane, and then immunoblotted for amelogenin Western blot analysis. **(K)** Densitometry of the Western blot in **(J)**.

To interrogate the effect of acidic pH on apatite nucleation and crystal growth, crystal growth studies with varying pH were conducted on polystyrene dishes and on carbon coated electron microscopy grids. On plastic dishes, apatite crystal growth at pH 4.0 vastly outperformed crystal growth at other pH values, reaching crystal dimensions of 1.5 mm length in average and exceeding those grown at pH 6.0 and 7.0 approximately 100-fold (Figures 4A–G). On electron microscopy grids, mineral assemblies formed at pH 4.0 were doubled in size and number when compared to those grown at pH 7.0 (Figures 4H,I). To determine whether there was a pH-dependent change in apatite binding, N92 N-terminal amelogenin proteins were adsorbed to apatite at varying pH values, and lysed proteins were compared *via* Western blot (Figures 4J,K). Densitometry demonstrated that amelogenin adsorption to apatite was greatest at pH 5 and pH 6 (Figure 4K), suggesting that amelogenin-apatite interactions changed with pH but did not appear to directly affect the dramatic changes in self-assembly and crystal growth detected in the other experiments conducted in this study. However, changes in amelogenin binding may also have other explanations.

### Enrichment of self-assembled enamel protein clusters with mineral ions in phosphate buffer at pH4 revealed grape cluster-shaped enamel protein assemblies as ion-laden reservoirs for initial enamel crystal growth

**FIGURE 5:**
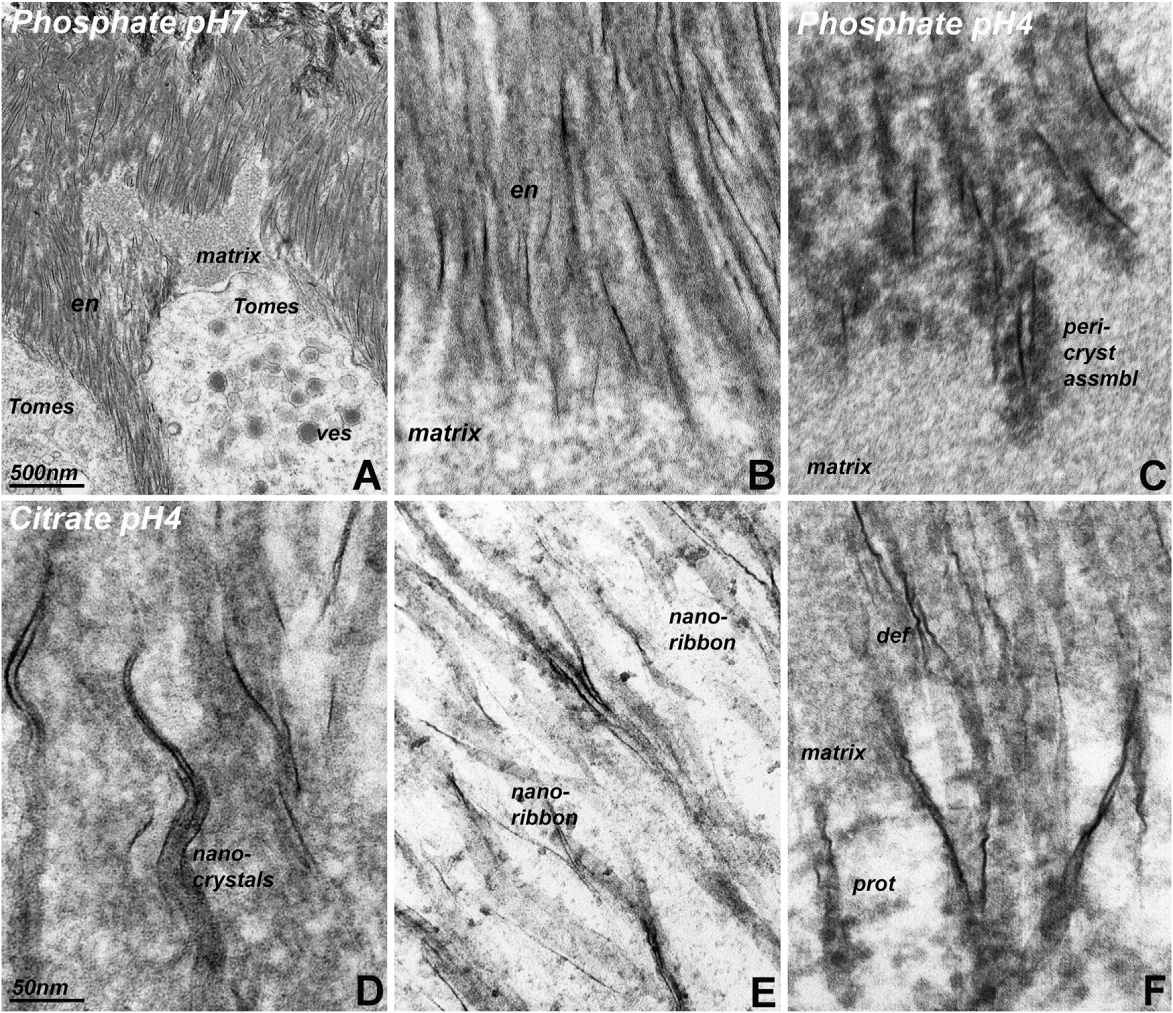
Transmission electron micrographs of initial enamel crystals embedded in enamel matrix after immersion in phosphate and citrate buffers at pH 4 and pH 7. **(A)** Overview electron micrograph illustrating the position of Tomes’ process (Tomes) loaded with secretory vesicles (ves) adjacent to enamel matrix deposits (matrix) and newly formed enamel crystals (en). **(B)** is a high magnification image of the initial enamel crystal (en)/matrix (matrix) interface of the same specimen. **(A,B)** were subjected to phosphate buffer at pH 7. **(C)** Initial enamel crystal/matrix interface in a specimen subjected to phosphate buffer at pH 4. Note the intensely contrasted peri-crystalline protein matrix (peri-cryst assembl) adjacent to the newly forming enamel crystals. **(D–F)**. Initial enamel crystal/matrix interface in a specimen subjected to citrate buffer at pH 4. The use of citrate buffer illustrates components of the initial enamel mineral phase, including nanocrystals (nanocrystals), ribbons (nanoribbon), and elongated enamel crystals). Protein assemblies (prot, **(F)** were less exposed than after immersion of the specimen in phosphate buffer. Note the presence of corkscrew-like crystal deformations (def). The scale bar in **(A)** only applies to **(A)**, while the scale bar in **(D)** applies to **(B–F)**.

To ask how highly acidic conditions affect initial enamel matrix and crystal growth environment, 3 days old postnatal mouse molars were immersed in two different buffers at pH 4.0 for 2 h and then subjected to fixation to preserve resulting ultrastructure. The ultrastructure of samples kept in phosphate buffer at pH 7.0 has been well characterized (Diekwisch et al., 1995) and featured secretory vesicles, secretory enamel matrix and nucleating enamel crystals (Figures 5A,B). However, at pH 4.0, crystals were surrounded by intensely stained clusters of berry-shaped assemblies, resembling overgrown drapes of nanospheres (Figure 5C). In contrast, samples treated with citrate buffer at pH 4.0 displayed bundles of initial enamel apatite nanoribbons (Figures 5D,E), and only few amelogenin supramolecular assemblies (nanospheres) aligned with the crystal bundles (Figure 5F). Studies presented here suggest that acid pH clusters are reflective of intermediate stages of nanosphere assemblies during crystal formation.

### AMEL and AMBN co-localized and interacted at pH 4 and pH 4.5

**FIGURE 6:**
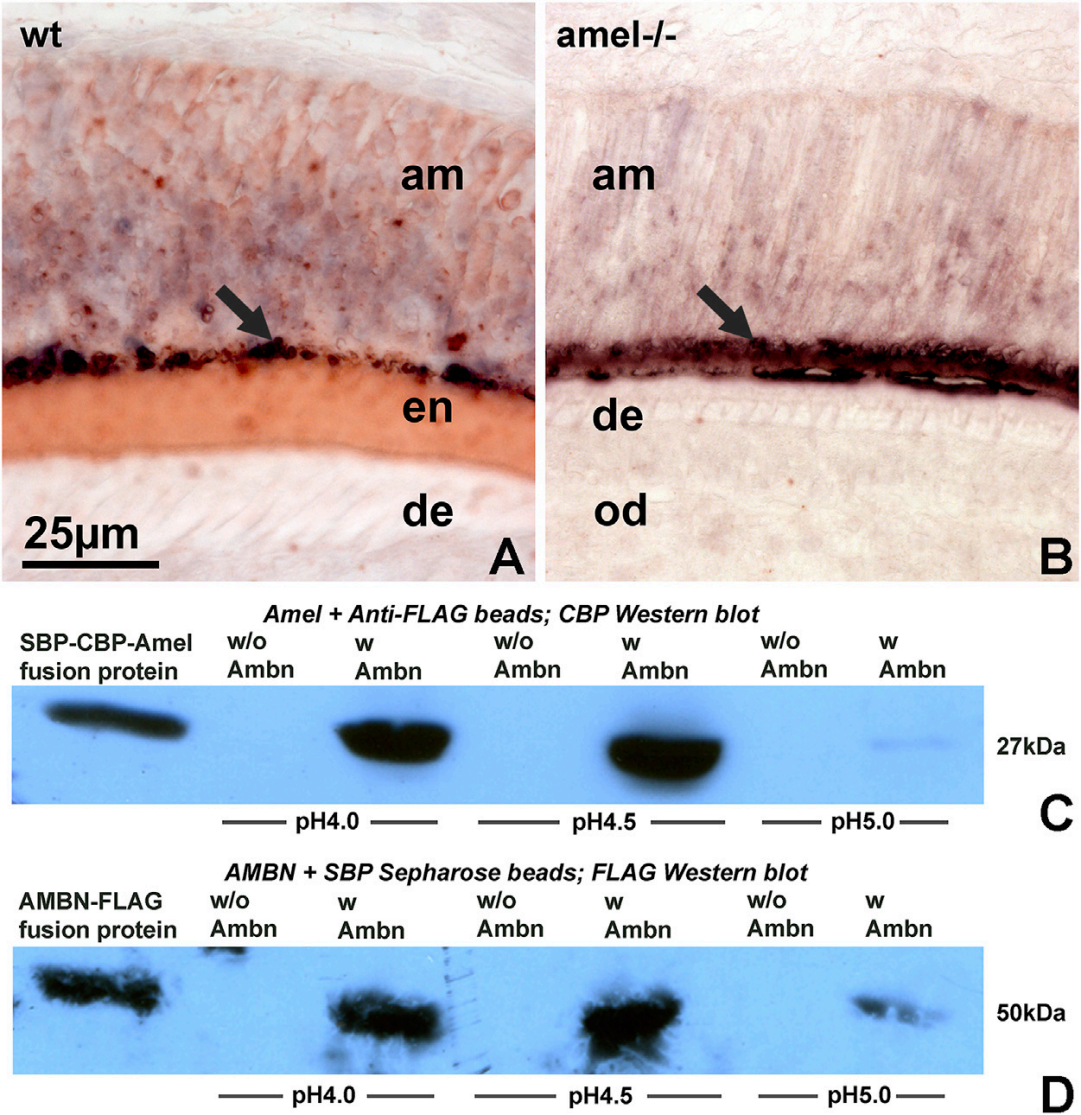
Amelogenin and ameloblastin enamel matrix molecules interact at acidic pH. **(A,B)** Double staining for amelogenin (HRP labeling, red color) and ameloblastin (AP labeling, blue/black color) on tissue sections of wild-type **(A)** and amelogenin null **(B)** molars. **(C,D)** Co-precipitation to detect possible interactions between amelogenin and ameloblastin at pH 4, pH 4.5, and pH5. Co-precipitates were either probed with anti-CBP (C, for amelogenin) or with anti-FLAG antibody (D, for ameloblastin). wt = wild-type, amel−/− = amelogenin mutant, am = ameloblasts, en = enamel, de = dentin, od = odontoblasts.

We then asked how acid conditions affect interactions between the two major enamel matrix proteins, amelogenin and ameloblastin. In developing 1-day old postnatal mouse molars, amelogenin was localized in the ameloblast layer and in the enamel, while ameloblastin was tightly concentrated at the ameloblast/enamel interface (Figure 6A). This tight concentration of ameloblastin at the ameloblast/enamel interface was retained in same stage molar organs of amelogenin null mice (Figure 6B). Co-immunoprecipitation studies were performed to determine how acidity and isoelectric point affected interactions between amelogenin N92 and ameloblastin (Figures 6C,D). Co-precipitates were either probed with anti-CBP to recognize SBP-CBP-Amelogenin fusion proteins or with anti-FLAG to recognize AMBN-FLAG fusion proteins generated in a separated experiment, and Western blots revealed strong amelogenin-ameloblastin interactions at pH 4.0 and 4.5, and only very weak interactions at pH 5.0 (Figs. C,D).

## Discussion

The present study was prompted by the detection of an extremely acidic pH 4 environment in the earliest secretory enamel matrix and examines its implications on enamel protein self-assembly, calcium phosphate and enamel crystal growth, and enamel protein interactions. Effects of acidic pH on amelogenin self-assembly were studied using circular dichroism (CD), attenuated total reflectance Fourier transform infrared (ATR-FTIR), and atomic force microscopy (AFM). Interactions between the amelogenin TRAP domain (AA1-45) and the N-terminal portion of LRAP (AA1-33) at pH 4.5 and pH 6 were tested using NMR and HSQC spectra. Calcium phosphate and apatite crystal growth studies at pH values between pH 3.0 and 8.0 were conducted either on polystyrene plates or on TEM grids, and binding of apatite to N92 amelogenin at pH values between pH 3.0 and 8.0 was analyzed on Western blots. Freshly dissected developing molar tooth organs were immersed in either phosphate or citrate buffer at pH 4 and 7, and the effect of these two buffers at pH 4 and 7 was compared. To examine the effect of acidic pH on interactions between two major enamel proteins, amelogenin and ameloblastin were co-localized at the ameloblast/initial enamel matrix interface, and amelogenin/ameloblastin binding at pH4, pH4.5, and pH5 was compared using co-immunoprecipitation. Finally, the C-terminal amelogenin/ameloblastin interaction domain was identified using NMR. Together, these studies provide evidence for a highly acidic microenvironment in the earliest secreted enamel matrix at the ameloblast secretory pole and characterize the effects of such an environment on amelogenin self-assembly, calcium phosphate crystal growth, and amelogenin/ameloblastin interactions.

pH indicator dyes revealed pH values between pH 3 to 4 at the exocytosis site of secretory ameloblasts. This was a remarkable finding as living organisms rarely endure extreme pH values. Exceptions are a few cyanobacteria which live in a pH 2.8–4.5 environment (Steinberg et al., 1998), the mucosal lining of the stomach which tolerates pH 1.5–2.0 gastric acid (Fujimori, 2020), and some of the acidic proteins involved in crystal nucleation such as dentin phosphoprotein (pH 2.67) (Marin and Luquet 2007; Alvares 2014). In our study, the acidic pH zone measured approximately 100 µm in thickness while the thinnest pH microelectrodes available to us were 1 mm in thickness (ThermoFisher Scientific, Carlsbad, CA), rendering pH detection with electrodes beyond detection capabilities (data not shown). Additional studies using quantifiable pH indicators (Damkier et al., 2014) might provide additional means to measure the precise pH of the enamel matrix at this stage. Previous studies have been based on extracted incisors devoid of cells and attached matrix. Studies here have allowed us to visualize the pH at the very narrow 20 um interface between ameloblasts and enamel. According to our micrographs, the zone of highly acidic pH was not only limited to the ameloblast/matrix interface but also included the secretory ameloblast vesicles, likely due to their content of inorganic monophosphate. The narrow confines of the low pH zone may be explained by the high buffering capacity of amelogenin proteins and the abundance of other buffer systems such as carbonate buffers in the early enamel matrix (Lacruz et al., 2010; Bronckers et al., 2012; Varga et al., 2018).

The presence of a thin layer of extremely acidic pH between the pH neutral ameloblasts and the otherwise fairly balanced enamel matrix begs the question whether there would be any functional advantages for such an unusually acidic interface during amelogenesis. Decades of amelogenin protein biochemistry have attested to the high degree of amelogenin solubility at pH 3 and pH 4 (Burgess and MacLaren 1965; Fincham 1968). The present study also has provided evidence for advantageous growth conditions for calcium phosphate and apatite crystals at pH 4. Furthermore, an acidic pH of 2.8 has proven advantageous for the synthesis of apatite crystals (Chen et al., 2006). Finally, electron micrographs presented in this study provide evidence for an enhanced capacity of the enamel crystal-associated enamel protein matrix to adsorb and store phosphate ions for enamel crystal growth. The extreme pH may also play a role in triggering the catalytic activity of MMP20 since metalloproteinases are known to be activated in a pH dependent fashion (Johnson et al., 2000). Amelogenin cleavage at acid pH would remove amelogenin’s highly soluble C-terminus, resulting in the formation of insoluble amelogenin assemblies, which then would guide enamel apatite crystal growth through particle attachment (Jokisaari et al., 2019; Pandya and Diekwisch 2021).

Previous studies have demonstrated that the developing enamel matrix is a highly dynamic microenvironment greatly influenced by physico-chemical conditions such as temperature and pH (Diekwisch et al., 1993). In some of these studies, samples were to be prepared at 4°C to avoid the self-assembly and nanosphere formation at 37°C, which were thought to be artifacts at that time (Lyaruu et al., 1982; Lyaruu et al., 1984). In terms of pH, many protein biochemistry and 3D NMR structure studies have been conducted at pH 3 or pH 4 to avoid amelogenin aggregation or self-assembly at neutral pH such as pH 7. As much as these studies have yielded meaningful amelogenin structural data, it has not been clear whether such data were relevant *in vivo* (Delak et al., 2009; Zhang et al., 2011). In the present study we are suggesting that the enamel matrix is secreted at a highly acidic pH during the transitional stage immediately after the exit of enamel proteins from the ameloblast secretory vesicles and prior to the broadening of the enamel matrix for crystal growth. Such acidic conditions would facilitate rapid crystal growth as demonstrated in our study and as described in earlier apatite synthesis studies (Chen et al., 2006) followed by another period of enamel protein matrix self-assembly. Once equilibrated through amelogenin and other buffers, the reconfigured enamel protein matrix would then further define enamel apatite crystal growth and assembly in c-axis direction (Jokisaari et al., 2019; Pandya and Diekwisch 2021).

Changes in pH greatly affected the histidine rich region of the amelogenin coil domain (H47-H69) while the non-LRAP portion of the TRAP domain (AA34-45) emerged as the key sequence motif for amelogenin self-assembly at pH 6. Already the earliest studies in enamel protein chemistry characterized amelogenins as proteins rich in proline and histidine (Eastoe 1979). While much attention has been paid to the abundance of prolines in the amelogenin sequence (Jin et al., 2009), the relatively high number of histidines in the amelogenin molecule has remained enigmatic. Histidine is an essential amino acid uniquely characterized by a side chain pKa close to physiological pH, rendering histidine-rich proteins highly susceptible to changes in environmental pH, which then result in tautomerization or ring flips to interconvert protonated and unprotonated nitrogens (Li and Hong 2011). As a result, histidines often act as molecular switches that introduce conformational changes from simple molecular bonds to large-scale β-sheet-based conformations (Valéry et al., 2015). In terms of amelogenin, changes in pH are bound to affect the protonation of the 13 histidines of the mouse amelogenin and especially of the histidine-rich amelogenin coil region. In a highly acidic environment such as the initial enamel matrix at pH 4, histidine sidechains would be positively charged, resulting in weak intermolecular interactions. When continuous buffering causes the pH to increase to pH 6, about half of the His sidechains become neutral and hydrophobic, resulting in enhanced inter-molecular interactions, which would explain the strong amelogenin self-assembly in the developing enamel matrix as its pH turns to pH6 and above. According to previous studies (Zhang et al., 2011), this self-assembly is mostly facilitated by the α-helix rich amelogenin N-terminus. Data presented here suggest that the non-LRAP portion of the TRAP domain (AA34-45) is the key region involved in the pH associated changes in amelogenin self-assembly. According to our study, increasing pH increased the diameter of self-assembled amelogenin subunits from 2 to 3 nm at pH 5 to approximately 20 nm at pH 7, with a unique elongation phase at pH 6. The small self-assembly subunits at pH 5 might facilitate the assembly of small mineral particles and promote apatite growth through small particle attachment (Jokisaari et al., 2019; Pandya and Diekwisch 2021), while the elongated amelogenin structures at pH 6 are ideally suited to promote crystal elongation.

Our circular dichroism data match the results of earlier studies, illustrating the shallowing of the trough from a globally disordered monomer at low pH to complex amelogenin assembles at physiological pH due to protein assembly (Goto et al., 1993; Lakshminarayanan et al., 2010). ATR-FTIR spectra demonstrated substantial changes from low pH to physiological pH. However, individual spectra differed from previously published spectra (Renugopalakrishnan et al., 1986; Beniash et al., 2012), likely due to differences in sample preparation and experimental setup.

Our data demonstrated that amelogenin and ameloblastin co-localized at the secretory ameloblast cell membrane/enamel matrix interface and strongly interacted at pH 4 and pH 4.5, but no longer at pH 5. Previous studies have demonstrated distinct enamel protein presence at the ameloblast cell membrane (Satchell et al., 2002) and documented that an ameloblastin-rich enamel matrix devoid of amelogenin favors short and randomly oriented apatite crystals (Lu et al., 2011). Ameloblastin has also been shown to interact with lipid membranes and large unilamellar vesicles similar to those found in Tomes’ process (Su et al., 2019). Together, these studies in conjunction with our data suggest that ameloblast ameloblastin plays a role in the adhesion of the functional enamel matrix to the ameloblast cell membrane and in the establishment of a proper environment for initial enamel crystal growth and habit.

## Data Availability

The original contributions presented in the study are included in the article/supplementary material, further inquiries can be directed to the corresponding authors.
